# The American Journal of Obstetrics and Diseases of Women and Children

**Published:** 1871-08

**Authors:** 


*The American Journal of Obstetrics and Diseases of Women and Children*, edited by B. F. Dawson, M.D., published quarterly in May, August, November, and February. With the present number (May, 1871), this journal enters upon its fourth volume, William Baldwin & Co., 21 Park Row, New York, have taken charge of its publication, and are issuing it in good form and style.

